# Changes in the Management of Malignant Bone Tumors in the COVID-19 Pandemic in Developing Countries

**DOI:** 10.7759/cureus.25245

**Published:** 2022-05-23

**Authors:** Vivek Tiwari, Pankaj Kumar Sharma, Venkatesan Sampath Kumar, Rishi R Poudel, Sanjay Meena, Roshan Banjara

**Affiliations:** 1 Department of Orthopedics, All India Institute of Medical Sciences, Nagpur, IND; 2 Department of Orthopedics, All India Institute of Medical Sciences, Bathinda, IND; 3 Department of Orthopedics, All India Institute of Medical Sciences, New Delhi, IND; 4 Department of Orthopedics, Nepal Cancer Hospital and Research Center, Lalitpur, NPL; 5 Department of Orthopedics, Lady Hardinge Medical College, New Delhi, IND

**Keywords:** coronavirus, clinical oncology, low-income countries, ewing’s sarcoma, osteosarcoma, bone neoplasms

## Abstract

The COVID-19 pandemic caused by severe acute respiratory syndrome coronavirus 2 (SARS-CoV-2) has drastically affected healthcare delivery to cancer patients, including those with malignant bone tumors, worldwide. Such cancer patients are more susceptible to COVID-19 infection and risk contracting the severe disease, but their holistic tumor management has also suffered a significant impact. Because of the acute shortage of healthcare resources due to their diversion in COVID management, substantial changes are needed in various aspects of management for high-grade tumor patients, particularly in developing countries and population-dense regions, so that their evidence-based appropriate treatment is ensured. Owing to a lack of consensus regarding the ideal course of action for the management of malignant bone tumors in the current situation, many such patients often get neglected, leading to loss of life/limb. This review elaborates on various guidelines proposed by different healthcare organizations and institutes regarding the modified care pathways for malignant bone neoplasms in the current coronavirus pandemic. The early published results of these modified care pathways and the changes in the oncology practice brought about by the pandemic are also discussed.

## Introduction and background

The COVID-19 pandemic due to the global and unprecedented spread of severe acute respiratory syndrome coronavirus 2 (SARS-CoV-2) severely affected the healthcare system and healthcare delivery worldwide. After relative containment owing to several remedial measures in the first wave, the second and third waves of this dreadful disease took heavy tolls on many countries, especially developing countries, including India. As of May 13, 2022, there have been more than 517 million cases of this disease worldwide with more than six million deaths [1].

The management of other life-threatening diseases such as cancers, including malignant bone tumors, has also taken a serious brunt due to the COVID-19 pandemic. Cancer patients are not only more vulnerable to COVID-19, but the severity of COVID-19 disease is also found to be higher in them [2]. A multicentric retrospective study of 205 cancer patients with COVID-19 infection from China reported 20% mortality, with hematological tumors having poorer outcomes than solid tumors [3]. Besides, morbidity and mortality are also increased in such patients due to the lack of timely and appropriate treatments for cancers. Firstly, due to travel restrictions imposed by national lockdowns, many patients are not able to avail of hospital services on time. Secondly, due to the perceived risk of the spread of the COVID-19 disease, particularly in healthcare settings, many oncologists modified their practice, with 9% of healthcare workers having completely stopped OPD and IPD services [4-6]. To streamline the holistic management of malignant bone tumors in the COVID-19 pandemic, many guidelines were proposed by different organizations. However, there is still a lack of consensus regarding the ideal course of action for the management of malignant bone tumors in such pandemic situations, particularly in resource-limited healthcare settings of the world.

The current literature review discusses the various proposed modifications in the management of malignant bone tumors, summarizing the recommendations, the published outcomes of the modified care pathways, and the changes observed in the orthopedic oncology practice due to the COVID-19 disease.

## Review

Search strategy and selection criteria

References for this review were searched on PubMed using the search terms “COVID,” “Coronavirus,” “SARS-CoV-2,” “bone tumors,” and “sarcoma.” Articles were also identified through references to the searched articles. Only articles published in English were reviewed. The final reference list was prepared on the basis of relevance to the broad scope of this review.

Referral to dedicated tertiary care oncology centers

To match the available resources with the demand for patients with bone tumors, those patients who require urgent discussion in multidisciplinary team (MDT)/tumor board meetings, urgent tissue diagnosis by biopsy, and prompt treatment for limb salvage/saving life need to be prioritized. Patients with benign tumors and those with bone lesions where suspicion of malignancy is not high can be managed by the local orthopedics in the current scenario, although the diagnosis of malignant tumors should not be missed [7,8]. Besides efficiently utilizing the limited resources, this practice will also help in decreasing patient numbers at tertiary care referral oncology centers and will help in maintaining social distancing to prevent the spread of COVID-19.

COVID-free hospitals

Restivo et al. first recommended setting up COVID-free hospitals for the management of cancer patients [9]. Considering the increased susceptibility of cancer patients to COVID-19 disease, the logic behind this recommendation was to create safe pathways for the management of cancer patients. The same rule applies to patients with malignant bone tumors as well. The early experience with a COVID-19-free facility for sarcoma management reported by the Scientific Institute for Research, Hospitalization and Healthcare (IRCCS) Regina Elena National Cancer Institute, Italy (a regional referral oncologic center), was very promising [10]. After this, COVID-free facilities were also set up in the UK for the management of cancer patients [11]. In the COVID-19 pandemic, the use of COVID-free dedicated orthopedic oncology centers should be encouraged to prevent COVID-related morbidity, particularly for malignant tumor patients.

Summary of Recommendation

Countries should focus on dedicated COVID-free hospitals for cancer care. These centers should work closely with a nearby COVID center to manage postoperative patients if they contract COVID-19 despite adequate precautions.

General preventive measures against COVID-19

Social distancing with the maintenance of at least 2 meters distance between two patients and between patients and hospital staff, the use of hand sanitizers frequently, and the use of masks, undoubtedly, form the threefold essential strategy in preventing the spread of COVID-19 infection. The use of appropriate and good-quality personal protective equipment (PPE), including N95 masks, gloves, face shields, coveralls, shoe covers, and hoods/caps in various combinations, depending on the clinical scenario, is mandatory to protect healthcare workers from the deadly virus. Adequate training of the staff in donning and doffing of PPE should be ensured. In addition, the visitors of patients in hospitals should be reduced to one only, and also, the time for such visits needs to be restricted to as minimum as possible. Swift and proper sanitization of places need to be done in the case of encounters with suspected/confirmed COVID-19 patients. Also, interdepartmental referrals should be strictly done through telephones, emails, or video calls [10].

Outpatient services

Oncologists from Italy advocated prescreening via phone interviews 1-2 days before the scheduled visit and also screening at the checkpoint of the hospital [10]. Among the new patients, only those with suspicion of malignant or locally aggressive benign lesions should be entertained for undergoing a thorough diagnostic workup. For the already-diagnosed cases, medical records can be evaluated one week before the visit, and those with malignant tumors need to be managed as per the usual protocol. All outpatient visits that are deemed nonessential in the current times need to be canceled. Instead, telephone/video consultations should be arranged for such patients. The Oxford bone tumor and soft tissue sarcoma service reported a reduction in the number of outpatients from 43 patients/week to eight patients/week after such policy changes [7]. Also, patients need to be called well in advance (at least 30 minutes to one hour) to avoid the formation of waiting lines, particularly in population-dense regions, and collect various investigations before the consultation. Moreover, a study of COVID-19-positive cancer patients from China recommended that stronger personal protective provisions should be made for cancer patients and cancer survivors as they are prone to catching the COVID-19 virus easily [2].

According to the European Society of Medical Oncology (ESMO) guidelines, patients with high priority for outpatient visits include those with any suspicion of sarcoma or with newly diagnosed intermediate-/high-grade sarcoma, operated follow-up patients who are clinically unstable in the postoperative period, and those having signs of relapse at follow-up [12]. The National Institute for Health and Care Excellence (NICE) guidelines from the UK for the delivery of systemic anticancer treatment also recommend reduction of face-to-face contact by various provisions, including using telephone/videos, reducing nonessential follow-ups, encouraging the use of local laboratory services for giving blood tests, establishing drive-through pickup points for medicines, and also providing home delivery for chemotherapeutic medications [13]. Experience during the early COVID times from a quaternary referral musculoskeletal oncology center in Montreal, Canada, recommended a triage model where routine follow-ups should be done through telemedicine by reviewing investigations through a well-established network for the relay of investigation reports, and immediate postoperative patients should be seen in clinics for wound inspections [8]. Also, splitting of clinic visit times was suggested in order to decrease the risk of exposure to the senior faculty members, with only one senior member being present in each session.

Summary of Recommendation

Physical outpatient visits need to be reduced, and teleconsultations should be encouraged. All face-to-face consultations need to be planned well in advance, with provisions for adequate and appropriate personal protective equipment.

Diagnostic facilities

Tumor diagnostic and staging workups entail ordering various investigations, including blood investigations; imaging studies that include X-rays, CT scans, and MRI; nuclear medicine investigations such as bone scan and PET scan; and the most crucial pathology services for providing biopsy results. In the current situation, there should be an extremely judicial ordering of required investigations both to save the limited resources and to prevent the spread of the disease by avoiding overcrowding. Blood investigations should be advised to be done at centers close to the patients’ homes, and unnecessary hospital travel should be mitigated [12]. Nuclear imaging investigations may not be readily available everywhere due to issues with the availability of radioisotopes. Teleradiology and telepathology services should be encouraged [14]. High-priority recommendations for various diagnostic facilities given by the Sarcoma European and Latin American Network (SELNET) and ESMO have been outlined in Table 1.

**Table 1:** High-Priority Recommendations for Diagnostic Facilities SELNET: Sarcoma European and Latin American Network; ESMO: European Society of Medical Oncology

SELNET [15]	ESMO [12]
New bone lesion having suspicion of malignancy	Diagnostic imaging studies (MRI/CT scan) to confirm the diagnosis for patients having suspected sarcomas
Those lesions having the risk of pathologic fracture	Biopsy to confirm the diagnosis (image-guided or clinically guided) for patients having suspected sarcomas
Giant cell tumor/osteochondroma suspected of malignant transformation	Diagnostic imaging studies, biopsy with pathology assessment, and echocardiography in patients having indication of anthracyclines, in cases of relapsed tumors
Those lesions having suspicion of local recurrence	Restaging studies in order to monitor the response to active treatments, although such studies can be done at relatively longer intervals
New bone tumors already having metastases	
Any new metastatic recurrence having abnormal behavior for the particular tumor	

Summary of Recommendation

Those patients having suspicion of bone malignancy should be prioritized for diagnostic imaging and biopsy services. Teleradiology and telepathology services should be encouraged.

Virtual MDTs

Bone sarcomas are a rare variety of cancers, constituting <1% of all malignant tumors in adults. However, due to the large heterogeneity in clinical presentation regarding the site, size, stage of disease, and age of presentation, decisions regarding the management of such tumors need to be made in MDTs/tumor board meetings. Since the beginning of this pandemic, it has been advocated to adopt virtual pathways for MDTs so as to avoid social gathering/crowding, innate to the prevention of COVID-19 spread. An online survey of practicing oncologists in Gulf and Arab countries revealed a high level of awareness regarding virtual modalities of management [4]. The use of such modern technology is also perceived to be associated with certain challenges, including the lack of physical attendance of patients precluding their physical examination, the safety of virtual management, and problems with Internet connectivity. Also, not many people in developing countries have access to various technologies in order to take benefit from virtual management. However, another online survey among the members of virtual tumor board meetings revealed very high satisfaction (73%) with the depth of discussion in such meetings [16]. Conduction of virtual MDTs has a unique advantage of involving faculties and experts from abroad for their input regarding appropriate decision-making in challenging cases.

Summary of Recommendation

Virtual MDTs should be adopted for decision-making regarding the management of bone sarcomas. The challenges associated with modern technology need to be adequately addressed.

Surgical management

Preoperative COVID-19 RT-PCR testing should be done for all patients undergoing surgery. If COVID-19 is suspected/confirmed, surgical management should be postponed until the symptoms are resolved to decrease life-threatening, serious complications [17,18]. A retrospective study of 1099 patients from China reported a 20.6% cumulative risk of ICU admission, need for mechanical ventilation, and death in COVID-19-positive patients undergoing surgery as compared to a 3.6% risk among the general COVID-19 patients [3]. Another multicenter study done on patients undergoing surgery with perioperative COVID-19 infection at 235 hospitals from 24 countries reported that more than half of these patients developed pulmonary complications with an overall 30-day mortality of 38% [19]. Besides, operating on COVID-19-positive patients is also perceived to be risky and dangerous for the operation theater staff. However, in a survey of 24 COVID-19-positive orthopedic surgeons from eight hospitals in China, Guo et al. reported that only 12.5% suspected infection exposure from the operating room, and all the surgeons recovered [20]. Moreover, a delay of even one week in surgery for osteosarcoma after neoadjuvant chemotherapy (NACT) is reported to increase the risk of recurrence by 1.14 times [21]. Therefore, curative surgery for high-grade bone sarcomas should not be delayed.

Operation lists need to be planned carefully, taking care of the aggressiveness of the tumor, the general condition of the patient, and the time elapsed since NACT; high priority should be given to cases where delay in surgery would cause danger to life/limb [7]. Adequate precautions need to be taken during all surgeries, including appropriately using level 2 PPEs, avoiding pulse lavage to reduce aerosol generation, and reducing traffic in the operation room [22]. Also, it has been recommended to postpone surgeries on elderly patients who are >60 years of age and those having ASA grade III/IV, owing to the higher risk of postoperative complications in this subset of patients [11]. Table 2 summarizes various high-priority recommendations for the surgical management of bone tumors in the COVID-19 pandemic.

**Table 2:** High-Priority Recommendations for the Surgical Management of Bone Tumors SELNET: Sarcoma European and Latin American Network; ESMO: European Society of Medical Oncology

Cardoso et al. [23]	IRCCS Regina Elena National Cancer Institute, Italy [10]	SELNET [15]	ESMO [12]
Sarcomas where surgery is first/unique curative treatment, such as chondrosarcoma	Biopsy in bone lesions with a suspicion of cancer and rebiopsy in cases where the previous biopsy was inconclusive	High-grade conventional osteosarcoma and chondrosarcoma	Ewing’s sarcoma
Surgery for sarcoma after NACT (osteosarcoma, Ewing’s sarcoma)	Curative resection of the biopsy-proven malignant bone tumor or its recurrence	Mesenchymal chondrosarcoma	Osteosarcoma
Recurrent sarcomas without any metastasis amenable to resection	Wide resection of malignant bone tumor planned for 3D printed/custom-made endoprosthesis	Skeletal Ewing’s sarcoma after NACT	Surgical complications of any type
Impending or established pathologic fracture of proximal hip region or spine with cord compression	Fixation of impending/actual pathologic fractures	Other high-grade primary bone tumors	Cases with discordant biopsy reports and that are suspected to be malignant
	Spine tumor with cord compression	Those with surgical complications	
	Management of any postoperative complications	High-grade/intermediate grade in local recurrence	
	Chondrosarcoma patients (as they are chemoresistant/radioresistant)	Metastatectomies as part of the multimodal management of osteosarcoma/Ewing’s sarcoma	
		Oligometastatic chondrosarcoma or other high-grade bone sarcomas without any evidence of local recurrence	

The availability of ICU facilities has been a serious issue due to their use for COVID-19 patients, and as a result, high-risk surgery such as malignant bone tumors involving the pelvis and sacrum may need to be deferred/postponed [22]. Some other challenges reported in these difficult times include the stoppage of frozen section services in some institutes for the risk of aerosol generation and implant unavailability owing to travel restrictions/lockdowns [22]. Often, however, these difficult situations entail a drastic change in the management of malignant bone tumors to save a life. Gaston et al. reported a case of osteosarcoma of the proximal humerus in a 17-year-old young patient, where limb salvage surgery was changed to forequarter amputation, as limb salvage was perceived to be associated with increased chances of nosocomial COVID-19 infection, would have required frequent follow-ups for a longer time, and would also have intraoperative difficulties including face mask visibility issues and heat stress owing to long-duration surgery [24].

Summary of Recommendation

Curative surgery of high-grade malignant bone tumors should not be delayed and be given the highest priority. Operation lists need to be planned carefully with a preference for younger patients (<60 years) and ASA grade I/II.

Chemotherapy

Certain modifications and adaptations have been suggested in chemotherapeutic treatments and regimens for malignant bone tumors [14]. A study of 1590 laboratory-confirmed COVID-19-positive cases from China reported that four patients who had a history of cancer and who had received chemotherapy/surgery within the last one month had a higher risk of severe events [2]. On the other hand, a retrospective observational study involving eight centers in the UK did not find any association between adjuvant chemotherapy and the risk of contracting the COVID-19 disease [25]. The authors from a COVID-free regional reference oncologic center in Italy reported starting “drug domiciliation projects” for oral chemotherapeutic medications in order to decrease hospitalization [10]. Table 3 summarizes the high-priority chemotherapy indications for bone sarcomas proposed by various organizations.

**Table 3:** High-Priority Chemotherapy Indications for Bone Tumors SELNET: Sarcoma European and Latin American Network; ESMO: European Society of Medical Oncology; NICE: National Institute for Health and Care Excellence

French Sarcoma Group [18]	SELNET [15]	ESMO [12]	NICE [13]
Continue NACT and adjuvant regimens as previously for osteosarcoma/Ewing’s sarcoma	NACT in osteosarcoma/Ewing’s sarcoma as well as potentially resectable mesenchymal chondrosarcoma	NACT/adjuvant chemotherapy for osteosarcoma/Ewing’s sarcoma	Curative treatment with >50% chances of success and NACT/adjuvant treatment adding at least 50% chance of cure to surgery/radiotherapy alone
Continue the standard treatment for primary bone sarcomas with metastases	Chemotherapy in recurrent advanced osteosarcoma/Ewing’s sarcoma and metastatic undifferentiated high-grade bone sarcoma	Limit the use of dexamethasone	
Topotecan and cyclophosphamide for Ewing’s sarcoma in cases of metastatic relapse	Upfront chemotherapy in metastatic osteosarcoma/Ewing’s sarcoma	Encourage the use of G-CSF/EPO growth factor and antibiotics in order to decrease the chances of neutropenia	
Antiangiogenic treatment for osteosarcoma in cases of metastatic relapse			

Delay should not be done in curative surgery for malignant bone tumors at a time dictated by NACT. Adjuvant chemotherapy for stable bone tumors can be delayed/postponed [2,7]. Among tumor patients on systemic chemotherapy, symptoms of COVID-19, neutropenic sepsis, and pneumonitis might be difficult to differentiate, and in cases of fever, neutropenic sepsis should be suspected and promptly treated as it can be life-threatening. In cancer patients with diagnosed COVID-19 infection, systemic anticancer treatment should be withheld for 10 days/until symptom resolution based on shared decision-making [13].

Summary of Recommendation

Certain modifications are suggested in chemotherapeutic regimens to decrease the risk of COVID-19 infection and delay surgery. However, curative surgery for malignant bone tumors should be done at the time dictated by NACT and should not be delayed.

Radiotherapy

The NICE UK has suggested arranging radiotherapy treatment for suspected/known COVID-19-positive patients at a particular time of the day and also arranging the schedules such that those with a higher risk of severe disease from COVID-19 infection are treated at a different time. Also, separate entry and exit areas should be ensured for different groups of patients [26]. Organizations such as NICE, SELNET, and ESMO have provided high-priority indications of radiotherapy for bone tumors, which are tabulated in Table 4.

**Table 4:** High-Priority Indications for Radiotherapy in Bone Tumors NICE: National Institute for Health and Care Excellence; ESMO: European Society of Medical Oncology; SELNET: Sarcoma European and Latin American Network

NICE [26]	ESMO [12]	SELNET [15]
Radical radiotherapy or chemoradiotherapy with curative intent, if treatment has already started and for those with category 1 (rapidly proliferating) tumors	Cases who are already in treatment	Skeletal Ewing’s sarcoma
External beam radiotherapy with brachytherapy, if it is already started and cases with category 1 tumors	Those with acute spinal cord compression, symptomatic brain metastases, or any other urgent palliative radiotherapy requirement	Definitive treatment of grade 3 chondrosarcoma
Those category 1 tumor cases who have not yet started treatment	Palliative treatment of bleeding/painful inoperable masses where symptoms cannot be resolved with medicines	Unresectable osteosarcoma after NACT
		Any symptomatic metastatic lesion where relief is expected with radiotherapy

In resource-constrained settings, cases with pelvic Ewing’s sarcoma may need to be provided definitive radiotherapy [22]. Hypofractionated radiotherapy regimens have also been suggested to decrease the number of hospital visits [10]. For malignant tumors involving the spine, radiotherapy has an important role in the definitive treatment and in delaying surgery [27].

Summary of Recommendation

Patients who are already on radiotherapy and those with rapidly proliferating tumors should be given the highest priority for continuing radiotherapy. Besides, radiotherapy has a significant role in palliative scenarios and in delaying surgery.

Clinical trials

COVID-19 has not only affected the initiation of new drug trials involving cancer patients but also negatively impacted the smooth continuation of ongoing trials. All complex decisions regarding clinical trials should be taken in virtual MDTs [18]. The SELNET suggests higher priority for new enrolment in clinical trials when the treatment is likely to improve the patient’s clinical outcome and to continue previous ongoing treatment if the patient is getting clinical/radiological benefit [15]. Similarly, the ESMO also provided high priority for the continuation of treatment in clinical trials [12].

Summary of Recommendation

New enrolment, as well as the continuation of those bone tumor patients who are already enrolled in clinical trials, should be contemplated when the treatment is likely to improve the patient’s clinical outcome.

Early results from the modified care pathways

Rossi et al. reported results of modified care practice from a COVID-free regional reference oncologic center in Italy where they treated 79 patients in the sarcoma unit over five months without any COVID-19-positive inpatient or healthcare worker identified [10]. Kumar et al. showed the results of 91 patients operated on for bone tumors over eight weeks, including 50 patients before national lockdown (four weeks) and 41 patients after lockdown (four weeks), at a tertiary bone sarcoma referral center in India [22]. They reported a significant increase in the number of major surgeries performed (24/50 versus 37/41, p<0.001) during lockdown owing to increased available operation theater time as other elective surgeries were postponed. They also did not obtain any positive cases among patients/staff after 15 days of follow-up. However, they could not operate on pelvic/sacral tumors owing to a lack of ICU availability. Stevenson et al. reported the results of 100 consecutive orthopedic oncology patients operated at a single center in the UK over around 2.5 months. They found five patients who were COVID-19-positive (four were on immunosuppressants preoperatively); three patients had pulmonary complications, and one patient died at a 30-day follow-up [17] (Table 5).

Rajasekaran et al. operated on 56 patients with bone and soft tissue tumors over two months at a tertiary center in the UK, 27 patients in an index hospital and 29 patients in a COVID-free facility [11]. They found that four patients had developed COVID-19, 13 had complications including pulmonary embolism in four patients and ARDS in four patients, and two patients died at 30-day follow-up. They found that the majority of complications occurred in ASA III/IV patients and that those aged <60 years and those operated at a COVID-free facility had fewer complications. In another retrospective multicenter study from eight centers in the UK, 347 patients with musculoskeletal tumors were operated on over around 2.5 months [25]. They reported that 12 patients (4%) developed COVID-19 disease, out of which four patients (1%) died. They also found that a higher ASA grade was associated with higher chances of getting COVID-19; however, adjuvant therapy was not found to be associated with COVID-19 (Table 5).

**Table 5:** Early Results From the Modified Care Pathways for Malignant Bone Tumors

Study name (duration)	Number of patients	COVID-19-positive patient/staff (n)	Follow-up (days)	Perioperative complications (n)
Rossi et al. [10] (December 1, 2019-April 30, 2020)	79	0	Not mentioned	Nil
Kumar et al. [22] (February 24-April 18, 2020)	91	0	15	(Non-COVID)
Stevenson et al. [17] (March 4-May 22, 2020)	100	5 patients	30	1 patient died, 3 had pulmonary complications, 1% 30-day mortality (adjusted: 0.6%)
Rajasekaran et al. [11] (March 12-May 12, 2020)	56	4 patients	30	2 patients died, 13 had complications, 4 had a pulmonary embolism, 3 had ARDS
Rajasekaran et al. [25] (March 12-May 20, 2020)	347	12 patients	30	4 patients died

Changes in oncology practice due to COVID-19

An electronic survey of practicing oncologists from Gulf and Arab countries in April 2020 reported a very high awareness of virtual management for cancers, including virtual clinics, virtual prescriptions, and virtual MDTs [4]. The majority of them believed that neoadjuvant, adjuvant, perioperative, and first-line palliative chemotherapeutic treatments should continue during the pandemic. On the other hand, a substantial proportion suggested stopping second-line and third-line palliative treatments.

Another online survey of 194 medical professionals, including 52% of orthopedic oncology surgeons, conducted in India in April 2020 revealed that 81% of participants modified their practice due to COVID-19 and that 10% completely stopped seeing patients [6]. A substantial proportion of surgeons (42%) avoided long-duration major surgeries, and 27% operated only in emergency cases. The majority of them (69%) preferred teleconsultation for follow-up patients. Yet another online survey of 149 musculoskeletal oncologists from the International Society of Limb Salvage (ISOLS) and European Musculo-Skeletal Oncology Society (EMSOS) also conducted in April 2020 reported that surgery for life-threatening sarcomas was stopped/delayed by around 40% respondents [5]. Around 20% of surgeons reported that patients were no longer followed up, and a similar proportion replied that postoperative physical therapy and rehabilitation services are not available for the patients. Moreover, two-thirds of the oncologists isolated themselves in various ways to stop the spread of COVID-19 infection to their family members. Thus, there have been drastic changes seen in the management of malignant bone tumors worldwide during the first wave of the COVID-19 pandemic, and we need to ensure that the healthcare requirements of such high-grade cancer patients are not overlooked in the future pandemic waves.

## Conclusions

The global COVID-19 pandemic caused by SARS-CoV-2 significantly affected patients with malignant bone tumors worldwide. The holistic management of these cancer patients needs to be ensured, especially in developing countries like India, and the associated morbidity and mortality should not be neglected. Modified care pathways, as recommended in the guidelines of various international organizations, need to be uniformly followed. The healthcare infrastructure should be promptly restructured to bring about appropriate changes required in the evidence-based management of bone tumors, particularly in resource-limited healthcare settings, in case of any future pandemic waves.

## References

[1] (2022). World Health Organization: WHO coronavirus (COVID-19) dashboard. https://covid19.who.int.

[2] Liang W, Guan W, Chen R (2020). Cancer patients in SARS-CoV-2 infection: a nationwide analysis in China. Lancet Oncol.

[3] Yang K, Sheng Y, Huang C (2020). Clinical characteristics, outcomes, and risk factors for mortality in patients with cancer and COVID-19 in Hubei, China: a multicentre, retrospective, cohort study. Lancet Oncol.

[4] Tashkandi E, Zeeneldin A, AlAbdulwahab A (2020). Virtual management of patients with cancer during the COVID-19 pandemic: web-based questionnaire study. J Med Internet Res.

[5] Thaler M, Khosravi I, Leithner A, Papagelopoulos PJ, Ruggieri P (2020). Impact of the COVID-19 pandemic on patients suffering from musculoskeletal tumours. Int Orthop.

[6] Gulia A, Tiwari A, Arora RS, Gupta S, Raja A (2020). Sarcoma care practice in India during COVID pandemic: a nationwide survey. Indian J Orthop.

[7] Rajasekaran RB, Whitwell D, Cosker TD, Gibbons CL (2020). Service delivery during the COVID-19 pandemic: experience from The Oxford Bone Tumour and Soft Tissue Sarcoma service. J Clin Orthop Trauma.

[8] Olshinka N, Mottard S (2020). Musculoskeletal oncology: patient triage and management during the COVID-19 pandemic. Curr Oncol.

[9] Restivo A, De Luca R, Spolverato G, Delrio P, Lorenzon L, D'Ugo D, Gronchi A (2020). The need of COVID19 free hospitals to maintain cancer care. Eur J Surg Oncol.

[10] Rossi B, Zoccali C, Baldi J (2020). Reorganization tips from a sarcoma unit at time of the COVID-19 pandemic in Italy: early experience from a regional referral oncologic center. J Clin Med.

[11] Rajasekaran RB, Kotecha S, Whitwell D (2020). Patient safety associated with the surgical treatment of bone and soft tissue tumours during the COVID-19 pandemic-results from an observational study at the Oxford Sarcoma Service. Int Orthop.

[12] (2022). European Society for Medical Oncology: ESMO‐EURACAN management and treatment adapted recommendations in the COVID‐19 era: Sarcomas. https://www.esmo.org/guidelines/cancer-patient-management-during-the-covid-19-pandemic/sarcomas-in-the-covid-19-era.

[13] (2021). National Institute for Health and Care Excellence: COVID‐19 rapid guideline: Delivery of systemic anticancer treatments. https://www.nice.org.uk/guidance/ng161.

[14] Gulia A, Arora RS, Panda PK (2020). Adapting management of sarcomas in COVID-19: an evidence-based review. Indian J Orthop.

[15] Martin-Broto J, Hindi N, Aguiar S Jr (2020). Sarcoma European and Latin American Network (SELNET) recommendations on prioritization in sarcoma care during the COVID-19 pandemic. Oncologist.

[16] Rajasekaran RB, Whitwell D, Cosker TD, Gibbons CL, Carr A (2021). Will virtual multidisciplinary team meetings become the norm for musculoskeletal oncology care following the COVID-19 pandemic? - experience from a tertiary sarcoma centre. BMC Musculoskelet Disord.

[17] Stevenson JD, Evans S, Morris G, Tillman R, Abudu A, Jeys L, Parry M (2020). Mortality of high-risk orthopaedic oncology patients during the COVID-19 pandemic: a prospective cohort study. J Surg Oncol.

[18] Penel N, Bonvalot S, Minard V (2020). French Sarcoma Group proposals for management of sarcoma patients during the COVID-19 outbreak. Ann Oncol.

[19] (2020). Mortality and pulmonary complications in patients undergoing surgery with perioperative SARS-CoV-2 infection: an international cohort study. Lancet.

[20] Guo X, Wang J, Hu D (2020). Survey of COVID-19 disease among orthopaedic surgeons in Wuhan, People's Republic of China. J Bone Joint Surg Am.

[21] Poudel RR, Tiwari V, Kumar VS, Bakhshi S, Gamanagatti S, Khan SA, Rastogi S (2017). Factors associated with local recurrence in operated osteosarcomas: a retrospective evaluation of 95 cases from a tertiary care center in a resource challenged environment. J Surg Oncol.

[22] Kumar VS, Banjara R, Thapa S (2020). Bone sarcoma surgery in times of COVID-19 pandemic lockdown-early experience from a tertiary centre in India. J Surg Oncol.

[23] Cardoso P, Rodrigues-Pinto R (2020). Surgical management of bone and soft tissue sarcomas and skeletal metastases during the COVID-19 pandemic. Eur J Surg Oncol.

[24] Gaston CL, Pag-Ong JP, Dacanay E, Quintos AJ (2020). Radical change in osteosarcoma surgical plan due to COVID-19 pandemic. BMJ Case Rep.

[25] Rajasekaran RB, Ashford RU, Cosker TD (2021). What proportion of patients with bone and soft tissue tumors contracted coronavirus-19 and died from surgical procedures during the initial period of the COVID-19 pandemic? Results from the multicenter British Orthopaedic Oncology Society observational study. Clin Orthop Relat Res.

[26] (2021). National Institute for Health and Care Excellence: COVID‐19 rapid guideline: Delivery of radiotherapy. https://www.nice.org.uk/guidance/ng162.

[27] Berjano P, Vanni D, Fariselli L, Cecchinato R, Boriani S (2020). Strategy for the practice of spine oncological surgery during the COVID-19 pandemic. Spine (Phila Pa 1976).

